# Cardiovascular and Noncardiovascular Prescribing and Mortality After Takotsubo: Comparison With Myocardial Infarction and General Population

**DOI:** 10.1016/j.jacadv.2023.100797

**Published:** 2024-01-10

**Authors:** Amelia E. Rudd, Graham Horgan, Hilal Khan, David T. Gamble, Jim McGowan, Arvind Sood, Ross McGeoch, John Irving, Jonathan Watt, Stephen J. Leslie, Mark Petrie, Chim Lang, Nicholas L. Mills, David E. Newby, Dana K. Dawson

**Affiliations:** a Aberdeen Cardiovascular and Diabetes Centre, University of Aberdeen and NHS Grampian, Aberdeen, United Kingdom; b Biomathematics & Statistics Scotland, Aberdeen, United Kingdom; c University Hospital Ayr, NHS Ayrshire and Arran, Ayr, United Kingdom; d Hairmyres Hospital, NHS Lanarkshire, East Kilbride, United Kingdom; e NHS Tayside, University of Dundee and Ninewells Hospital, Dundee, United Kingdom; f NHS Highland, Raigmore Hospital, Inverness, United Kingdom; g Institute of Cardiovascular and Medical Sciences, University of Glasgow, Glasgow, United Kingdom; h Usher Institute, University of Edinburgh, Edinburgh, United Kingdom; i Centre for Cardiovascular Science and Usher Institute, University of Edinburgh and NHS Lothian, Edinburgh, United Kingdom

**Keywords:** cardiovascular, electronic data linkage, medication, mortality, myocardial infarction, takotsubo

## Abstract

**Background:**

Takotsubo syndrome is an increasingly common cardiac emergency with no known evidence-based treatment.

**Objectives:**

The purpose of this study was to investigate cardiovascular mortality and medication use after takotsubo syndrome.

**Methods:**

In a case-control study, all patients with takotsubo syndrome in Scotland between 2010 and 2017 (n = 620) were age, sex, and geographically matched to individuals in the general population (1:4, n = 2,480) and contemporaneous patients with acute myocardial infarction (1:1, n = 620). Electronic health record data linkage of mortality outcomes and drug prescribing were analyzed using Cox proportional hazard regression models.

**Results:**

Of the 3,720 study participants (mean age, 66 years; 91% women), 153 (25%) patients with takotsubo syndrome died over the median of 5.5 years follow-up. This exceeded mortality rates in the general population (N = 374 [15%]; HR: 1.78 [95% CI: 1.48-2.15], *P* < 0.0001), especially for cardiovascular (HR: 2.47 [95% CI: 1.81-3.39], *P* < 0.001) but also noncardiovascular (HR: 1.48 [95% CI: 1.16-1.87], *P* = 0.002) deaths. Mortality rates were lower for patients with takotsubo syndrome than those with myocardial infarction (31%, 195/620; HR: 0.76 [95% CI: 0.62-0.94], *P* = 0.012), which was attributable to lower rates of cardiovascular (HR: 0.61 [95% CI: 0.44-0.84], *P* = 0.002) but not noncardiovascular (HR: 0.92 [95% CI: 0.69-1.23], *P* = 0.59) deaths. Despite comparable medications use, cardiovascular therapies were consistently associated with better survival in patients with myocardial infarction but not in those with takotsubo syndrome. Diuretic (*P* = 0.01), anti-inflammatory (*P* = 0.002), and psychotropic (*P* < 0.001) therapies were all associated with worse outcomes in patients with takotsubo syndrome.

**Conclusions:**

In patients with takotsubo syndrome, cardiovascular mortality is the leading cause of death, and this is not associated with cardiovascular therapy use.

Takotsubo syndrome (broken heart syndrome, stress-induced cardiomyopathy) is a potentially fatal cardiac emergency that mimics myocardial infarction in its presentation. It predominantly affects middle-aged women^1^ and is often triggered by mental or physical stress.^2^^,^^3^ Takotsubo patients have unobstructed coronary arteries and transient severe acute left ventricular dysfunction with myocardial “ballooning” that spontaneously recovers with restoration of normal left ventricular ejection fraction within days to weeks^4^ in the absence of any myocardial infarction/fibrosis. Despite this, several large registries have shown that the long-term prognosis of patients with takotsubo syndrome is comparable to that of patients with acute myocardial infarction.^5^, ^6^, ^7^ However, the specific causes of this increased mortality are currently unknown. Moreover, there is major uncertainty and heterogeneity of practice in the prescribing of medications after takotsubo syndrome, both in the type and duration of therapy. The impact of long-term cardiovascular and noncardiovascular therapies in takotsubo syndrome remains unknown.

Scotland has a unique and comprehensive national electronic health care resource that links primary, secondary, and tertiary care provision going back to 1986. We used the clinical outcomes and prescribing data from this resource to investigate the specific causes of mortality after takotsubo syndrome by comparing them with 2 contemporaneous age, sex, and geographically matched control groups: representative individuals from the general Scottish population and patients with acute myocardial infarction.

## Methods

### Study population

From January 1, 2010 to December 31, 2017, all patients diagnosed in Scotland with takotsubo syndrome (or with any known equivalent disease definition: acute stress cardiomyopathy, takotsubo syndrome or cardiomyopathy, apical ballooning)^8^^,^^9^ and fulfilling diagnostic criteria as per contemporaneous guidelines at the time of their index hospital presentation^8^^,^^9^ were included in the Scottish Takotsubo Registry (NCT03299569). This registry was approved by the National Research Ethics Committee as well as the Public Benefit and Patient Privacy Panel and was conducted in accordance with the Declaration of Helsinki.

In Scotland, all residents are allocated a unique Community Health Index number which is linked to all health care episodes: attendances to primary, secondary, or tertiary care centers, all medication prescriptions (which are reissued in primary care every 2 months), and all deaths. Because takotsubo syndrome did not have a specific code in the International Classification of Diseases-10th Revision (ICD-10) during the study period, after consultation with the research analysts curating the Information Services Division at Public Health Scotland we searched all health care records associated with an episode coded as I42.8, I42.9, and I51.8 (“other rare cardiomyopathies,” “cardiomyopathy, unspecified,” and “other ill-defined heart diseases,” respectively) in ICD-10. Independent adjudication of all 4,065 returns under the 3 ICD-10 codes was undertaken by researchers and the principal investigator at each health board for satisfying contemporaneous diagnostic criteria.^8^^,^^9^ In addition, and as a back-up verification, a further systematic inspection of all coronary angiography reports of patients who underwent urgent or emergency cardiac catheterization (n = 78,477) and did not proceed to percutaneous intervention or surgery (n = 8,205) were inspected.

### Study control populations

For each incident takotsubo syndrome case, a cohort of contemporaneous patients with acute myocardial infarction from the High-STEACS (High-Sensitivity Troponin in the Evaluation of Patients With Acute Coronary Syndrome; NCT01852123) trial database^10^^,^^11^ were age- and sex-matched in a ratio of 1:1 in each Scottish Health Board. This comprised consecutive patients presenting with suspected acute coronary syndrome to secondary or tertiary care hospitals in Scotland in whom the diagnosis of myocardial infarction was adjudicated according to the Universal Definition of Myocardial Infarction.^12^ Similarly, individuals from the Scottish general population were randomly selected by computer allocation within the electronic Data Research and Innovation Service branch of the Information Services Division at Public Health Scotland and were matched for age, sex, and geographic distribution of domicile (up to 50-mile radius in the most remote areas of Scotland) in a ratio of 1:4. Matching of the control populations was performed for sex and age (within 1 year) using nearest neighbor matching with R package MatchIT: Nonparametric Preprocessing for Parametric Causal Inference, Version 4.4.0.

All subsequent rehospitalization and death outcomes and repeated prescribing during the entire follow-up duration were obtained after electronic data linkage at Public Health Scotland, following which, all data were anonymized and made available to the researchers for analyses in the National Safe Haven environment of Public Health Scotland.

### Baseline clinical characteristics

Standardized demographic and clinical information were collected from each Health Board. Age, sex, past medical history, medication on admission, smoking status, and clinical presentation including heart rate and rhythm, blood pressure, 12-lead electrocardiography, routine cardiac biomarkers, echocardiography, coronary angiography, and left ventriculography were extracted from clinical records. Clinical characteristics of patients with myocardial infarction previously collected by the High-STEACS (NCT01852123) investigators^10^^,^^11^^,^^13^ were selected to match the predefined clinical characteristic categories of the takotsubo syndrome cohort as best as possible.

Baseline demographic characteristics of the controls identified from the general population are not available as firstly, these subjects do not have an index acute admission and secondly, they were selected centrally by Public Health Scotland rather than through their primary or secondary care health care provider, therefore their identity cannot be disclosed to the investigators.

### Clinical follow-up

For all 3 cohorts, information was extracted by electronic data linkage of routinely collected outcomes for each subject including all deaths and specific causes of death (as reported by the medical certificate of cause of death), medical and surgical secondary care admissions and diagnoses (as issued by the attending physician/surgeon), and all hospital and community medication prescribing, registered as dispensed to patient. A data dictionary was elaborated to facilitate the overall analysis and interpretation of data from the 3 populations.

Specific major causes of death were clustered into 17 major groups (Supplemental Table 1). Cardiovascular medications (angiotensin-converting enzyme inhibitor, angiotensin receptor blocker, beta-blocker, antiplatelet, statin, and diuretic therapies) and common noncardiovascular medications (anti-inflammatory drugs [steroidal and nonsteroidal], psychotropic medication, hormone replacement therapy, and thyroxine) were identified through prescribing databases. The highest level of prescription recording accuracy was used, that of prescription dispensed by pharmacy to patient for claim submitted monthly to National Health Service Scotland for reimbursement, as the best available surrogate of medication adherence for all medications. All data were anonymized before analysis in the Scottish National Safe Haven.

### Statistical analysis

Continuous variables are presented as mean ± SD, median (IQR) as appropriate, and categorical variables as counts (percentages). The Student’s *t*-test was used to compare continuous variables between groups and the chi-square test was used for categorical variables.

Outcomes were analyzed with Cox proportional hazards regression models and compared with Kaplan-Meier cumulative event curves. The proportional hazards assumption was assessed by calculating the correlation between scaled Schoenfeld residuals with time. Analyses were performed for the total follow-up period as well as a landmark analysis at 30 days to account for acute early events. When examining any specific cause of death (such as cardiovascular death), all other causes of death were treated as competing risks. Patterns were similar when Fine-Gray models were used. Associations with drug prescribing were analyzed with Cox regression for the total follow-up period and investigated according to whether each therapy had been prescribed at any time or prescribed for the majority (at least 50%) of the follow-up period. Age and sex were included as covariates. Data are presented as HRs with 95% CIs. For the analysis of takotsubo vs myocardial infarction, the myocardial infarction cohort was used as reference category. Chi-square tests were performed to assess the differences in treatment assignment based upon baseline characteristics. Variables that were significant were included in the adjusted Cox analysis separately for takotsubo syndrome (age, sex, electrocardiogram presentation, left ventricular (LV) ejection fraction, and coronary artery disease) and myocardial infarction (age, sex, electrocardiogram presentation, and coronary artery disease) to balance for potential confounders. Versions with and without diuretic use were obtained. Forest plots were constructed to display the estimated hazard ratios with each type of cardiac and noncardiac medications.

Because there was no adjustment for multiple testing in the analysis of endpoints, *P* values should be interpreted cautiously, except when they are very small (eg, *P* < 0.01) or consistent across several different related analyses. All analyses were performed with the use of R software, version 3.4.3 (R Foundation for Statistical Computing) and IBM SPSS Software, Version 27.

## Results

Overall, 743 hospital admissions were identified as patients with takotsubo syndrome. After removal of duplicates, readmissions and those lost to electronic follow-up, 620 were included as the final study population (Supplemental Appendix). Patient groups (n = 620 each) and the general Scottish population control subjects (n = 2,480) were well matched with a mean age of 66 years and 91% were women (Table 1). Clinical characteristics specific to the takotsubo syndrome patients are described in Table 2. Follow-up of all groups was censored on May 31, 2021. The median follow-up time was 5.5 years or 1,988 days (IQR: 1,460-2,705 days).

**Table 1:** Baseline Characteristics of the Patient Populations

	Patients With Takotsubo Syndrome (n = 620)	Patients With Myocardial Infarction (n = 620)
Demographics		
Female	564 (91%)	564 (91%)
Age, y	66 ± 12	66 ± 12
Body mass index, kg/m^2^	26 ± 5	30 ± 7
Leading trigger		
Emotional stress	247 (40%)	
Physical trauma	94 (15%)	
Concurrent medical/surgical illness	162 (26%)	
No stressor identified/not declared	117 (19%)	
Cardiovascular risk factors		
Hypertension	242 (38%)	246 (39%)
Diabetes mellitus	84 (13%)	111 (18%)
Hyperlipidemia	67 (11%)	230 (37%)
Smoking		
Current smoker	149 (29%)	211 (34%)
Ex-smoker	132 (26%)	97 (16%)
Nonsmoker	228 (45%)	146 (24%)
Alcohol intake	223 (55%)	
System comorbidities		
Cardiac^a^	172 (27%)	295 (48%)
Cancer	79 (13%)	
Respiratory	163 (26%)	
Neurological^b^	70 (11%)	
Autoimmune	66 (11%)	
Inflammatory	22 (4%)	
Gastrointestinal	160 (25%)	
Renal	73 (12%)	
Thyroid	88 (14%)	
Psychiatric illness	176 (28%)	
Anxiety	90 (14%)	
Depression	123 (20%)	
Schizophrenia	2 (0.3%)	
Bipolar disorder	3 (1%)	
Personality disorder	5 (1%)	
Physical suicide attempt	10 (2%)	
Overdose	17 (3%)	
Medication on presentation		
Aspirin	94 (15%)	210 (34%)
Beta-blocker	75 (12%)	161 (26%)
Calcium-channel blocker	23 (4%)	81 (13%)
ACE inhibitor/ARB	85 (14%)	211 (34%)
Statin	115 (18%)	230 (37%)
Diuretic	56 (9%)	100 (16%)
Primary symptoms on presentation		
Chest pain	469 (76%)	529 (85%)
Breathlessness	49 (8%)	30 (5%)
Collapse	27 (4%)	12 (2%)
Out-of-hospital arrest	9 (2%)	4 (1%)
Arrhythmia	2 (1%)	7 (1%)
Heart rate, beats/min	87 ± 22	82 ± 29
Systolic blood pressure, mm Hg	131 ± 30	132 ± 43
Diastolic blood pressure, mm Hg	80 ± 18	64 ± 36
ECG at presentation		
ST-segment elevation	259 (42%)	191 (31%)
Non-ST-segment elevation	258 (42%)	266 (43%)
Arrhythmia	23 (4%)	5 (1%)
Left bundle branch block	20 (3%)	2 (0.3%)
Normal	60 (10%)	144 (23%)
Troponin I (factor increase from upper limit of normal)^c^		
Admission troponin	134 ± 549	169 ± 602
12-h troponin	193 ± 1,023	679 ± 1,150
Coronary angiography		
Normal coronary arteries	550 (89%)	13 (3%)
Coronary disease present	70 (11%)	607 (97%)

Values are n (%) or mean ± SD.

ACE = angiotensin-enzyme converting; ARB = angiotensin receptor blocker; ECG = electrocardiogram.

a Cardiac—any coronary artery disease, arrhythmia, any valvular disease, inherited cardiomyopathy.

b Neurological—eg, disorders of the brain, spine, and nervous system. Parkinson disease, epilepsy, stroke, dementia, Huntington disease.

c Troponin I—expressed as factor increase above upper limit of normal in each Health Boards as different assays are used in different Health Boards.

**Table 2:** Clinical Characteristics of Takotsubo Syndrome Patients (N = 620)

Takotsubo syndrome type	
Apical	607 (98%)
Mid-ventricular	7 (1%)
Basal	4 (0.6%)
Focal	2 (0.3%)
Echocardiogram	
Left ventricular function	
Normal	84 (17%)
Mild impairment	92 (18%)
Moderate impairment	209 (41%)
Severe impairment	123 (24%)
Highest level of care	
Coronary care unit	146 (24%)
High dependency unit	5 (1%)
Intensive therapy unit	29 (5%)
Cardiology ward	440 (71%)
In-hospital outcome	
Cardiac device implantation^a^	18 (3%)
Cardiogenic shock	10 (2%)
LV thrombus	6 (1%)
Arrhythmia	4 (1%)
Death	15 (2%)

Values are n (%).

a Cardiac devices include implantable cardioverter defibrillators (ICD), implantable cardiac resynchronization therapy defibrillators (CRT-D), and permanent pacemakers (PPM).

### Total, cardiovascular and noncardiovascular mortality

**Figure 1:**
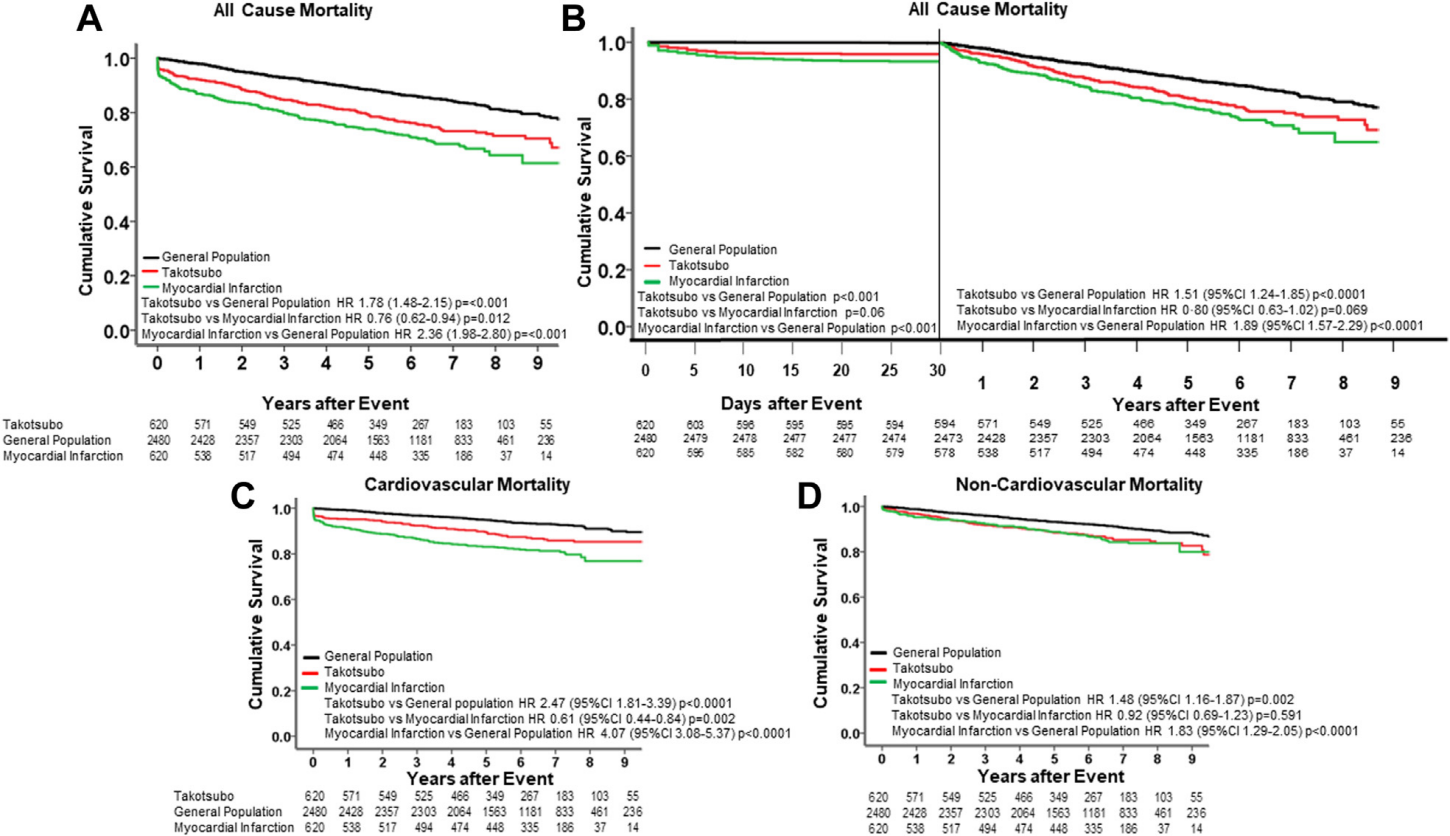
**All**-**Cause Mortality** (A) All-cause mortality in patients with takotsubo syndrome (red), acute myocardial infarction (green) and the general population (black). (B) Early (30-day) (left panel) and landmark post 30 days (right panel) all-cause mortality. Specific causes of mortality: cardiovascular (C), noncardiovascular (D).

Overall, 722 deaths occurred during the study follow-up period: 153 in patients with takotsubo syndrome, 195 in those with acute myocardial infarction, and 374 in the general population cohort. All-cause mortality was higher in patients with takotsubo syndrome compared to the general population (HR: 1.78; 95% CI: 1.48-2.15; *P* < 0.001) and slightly lower when compared to patients with acute myocardial infarction (HR: 0.76; 95% CI: 0.62-0.94; *P* = 0.012) (Figure 1A, Table 3). Similar patterns were seen for outcomes in the first 30 days and those beyond 30 days (Figure 1B). There was no difference in all-cause mortality in patients with takotsubo syndrome with coincidental coronary artery disease and those with unobstructed coronary arteries (HR: 1.05; 95% CI: 0.63-1.75; *P* = 0.852). Patients with takotsubo syndrome had higher rates of cardiovascular death compared to the general population (HR: 2.47; 95% CI: 1.81-3.39; *P* < 0.0001) but lower rates compared to patients with myocardial infarction (HR: 0.61; 95% CI: 0.44-0.84; *P* = 0.002) (Figure 1C, Supplemental Table 2). In contrast, noncardiovascular mortality was similar between patients with takotsubo syndrome and those with myocardial infarction (HR: 0.92; 95% CI: 0.69-1.23; *P* = 0.59), with both patient populations sustaining higher risks of noncardiovascular death compared to the general population (HR: 1.48; 95% CI: 1.16-1.87, and HR: 1.83; 95% CI: 1.29-2.05, respectively, *P* ≤ 0.002 for both) (Figure 1D).

**Table 3:** Specific Causes of Death

Cause of Death	Takotsubo Syndrome (n = 620)		Myocardial Infarction (n = 620)		General Population (n = 2,480)		HR (95% CI) Takotsubo vs General Population	*P* Value	HR (95% CI) Myocardial Infarction vs General Population	*P* Value	HR (95% CI) Takotsubo vs Myocardial Infarction^a^	*P* Value
	Number of Events/N	Event Rate, %	Number of Events/N	Event Rate, %	Number of Events/N	Event Rate, %						
All-cause death	153/620	24.7	195/620	31.5	374/2,480	15.1	1.77 (1.47-2.14)	<0.0001	2.32 (1.95-2.76)	<0.0001	0.76 (0.62-0.94)	0.012
Cardiac	43/620	6.9	86/620	13.9	66/2,480	2.7	2.69 (1.83-3.94)	<0.0001	5.51 (3.99-7.60)	<0.0001	0.49 (0.34-0.71)	0.0001
Gastrointestinal	5/620	0.8	2/620	0.3	7/2,480	0.3	2.86 (0.91-9.00)	0.0723	1.13 (0.23-5.60)	0.8783	2.53 (0.47-13.49)	0.2788
Infectious	12/620	1.9	12/620	1.9	44/2,480	1.8	1.14 (0.60-2.17)	0.683	0.93 (0.47-1.85)	0.837	1.23 (0.54-2.81)	0.6269
Neurological	3/620	0.5	11/620	1.8	8/2,480	0.3	1.50 (0.40-5.64)	0.5533	5.31 (2.17-12.97)	0.00025	0.28 (0.08-1.01)	0.051
Vascular (peripheral)	4/620	0.7	2/620	0.3	10/2,480	0.4	1.60 (0.50-5.09)	0.427	0.75 (0.16-3.41)	0.706	2.14 (0.39-11.74)	0.3806
Natural/accident	4/620	0.7	3/620	0.5	11/2,480	0.4	1.45 (0.47-4.54)	0.522	1.07 (0.30-3.78)	0.918	1.36 (0.31-6.01)	0.687
Psychiatric	2/620	0.3	2/620	0.3	5/2,480	0.2	1.59 (0.31-8.21)	0.578	1.49 (0.31-7.10)	0.620	1.07 (0.16-7.15)	0.943
Pulmonary	18/620	2.9	10/620	1.6	20/2,480	0.8	3.64 (1.93-6.87)	<0.0001	1.93 (0.91-4.10)	0.0884	1.89 (0.87-4.09)	0.1085
Renal			4/620	0.7	4/2,480	1.6			4.02 (1.01-16.04)	0.04919		
Endocrine	4/620	0.7	15/620	2.4	5/2,480	0.2	3.20 (0.87-11.89)	0.0826	11.65 (4.21-32.22)	<0.0001	0.27 (0.09-0.83)	0.0224
Cancer	34/620	5.5	30/620	4.8	113/2,480	4.6	1.21 (0.83-1.78)	0.323	1.02 (0.68-1.52)	0.926	1.19 (0.73-1.94)	0.486
Cerebrovascular	15/620	2.4	10/620	1.6	29/2,480	1.2	2.08 (1.11-3.87)	0.0214	1.34 (0.65-2.75)	0.4246	1.55 (0.70-3.45)	0.2821
Hemorrhage	1/620	0.2			2/2,480	0.1						
Chronic inflammatory					1/2,480	0.04						
Iatrogenic	1/620	0.1			1/2,480	0.04						
Dementia	7/620	1.1	8/620	1.3	48/2,480	1.9	0.58 (0.26-1.28)	0.1763	0.63 (0.30-1.34)	0.2302	0.91 (0.33-2.52)	0.8615

Event rates are based upon Kaplan-Meier estimates and are expressed as number of events per population and percentages. There was no adjustment for multiplicity in the analysis and results are reported as point estimates and 95% CI.

a For the analysis of Takotsubo syndrome (n = 620) vs myocardial infarction (n = 620), myocardial infarction was used as the reference group.

Among the 17 causes of death, pulmonary causes (n = 18) were the next most strongly associated with takotsubo syndrome (HR: 3.63; 95% CI: 1.92-6.87; *P* < 0.001 vs general population) with similar trends for patients with myocardial infarction (HR: 1.99; 95% CI: 0.92-4.31; *P* = 0.076 vs general population controls). There were no other causes of death (including cancer and dementia) associated with takotsubo syndrome (Table 3).

### Cardiovascular and noncardiovascular medications and mortality

The prescription rates of cardiovascular and noncardiovascular medication were similar between patients with takotsubo syndrome and patients with acute myocardial infarction whether analyzed either as medications prescribed at any time during follow-up or prescribed for the majority (at least 50%) of time during follow-up (Table 4). There were no major differences in the baseline characteristics of takotsubo syndrome patients who were prescribed cardiovascular medications or not during follow-up, except that older takotsubo syndrome patients appeared to receive more diuretic and patients with coronary artery disease present were more readily prescribed statin and antiplatelet therapy (Supplemental Table 3).

**Table 4:** Rates of Prescribed Cardiovascular and Noncardiovascular Medications

	Prescription Rate (%)	
	Patients With Takotsubo Syndrome (n = 620)	Patients With Myocardial Infarction (n = 620)
Cardiovascular medications		
Prescribed any time during follow-up		
ACE inhibitor/ARB therapy	80.7	78.4
Beta-blocker therapy	76.6	70.5
Antiplatelet therapy	71.3	86.3
Statin therapy	68.4	82.6
Diuretic therapy	43.7	50.0
Prescribed for majority (at least 50%) of follow-up time		
ACE inhibitor/ARB therapy	49.8	56.9
Beta-blocker therapy	46.1	51.0
Antiplatelet therapy	42.1	64.7
Statin therapy	46.8	61.8
Diuretic therapy	16.3	29.5
Noncardiovascular medications		
Prescribed any time during follow-up		
Steroids/NSAID therapy	57.4	53.2
Psychotropic therapy	66.9	62.4
Hormone replacement therapy	12.7	8.4
Thyroxine therapy	16.5	20.0
Prescribed for majority (at least 50%) of follow-up time		
Steroids/NSAID therapy	21.8	17.1
Psychotropic therapy	33.4	27.6
Hormone replacement therapy	10.7	6.5
Thyroxine therapy	13.9	16.3

Values are %.

ACE = angiotensin-converting enzyme; ARB = angiotensin receptor blocker; NSAID = nonsteroidal anti-inflammatory drugs.

#### Prescribing recorded at any time during follow-up

**Figure 2:**
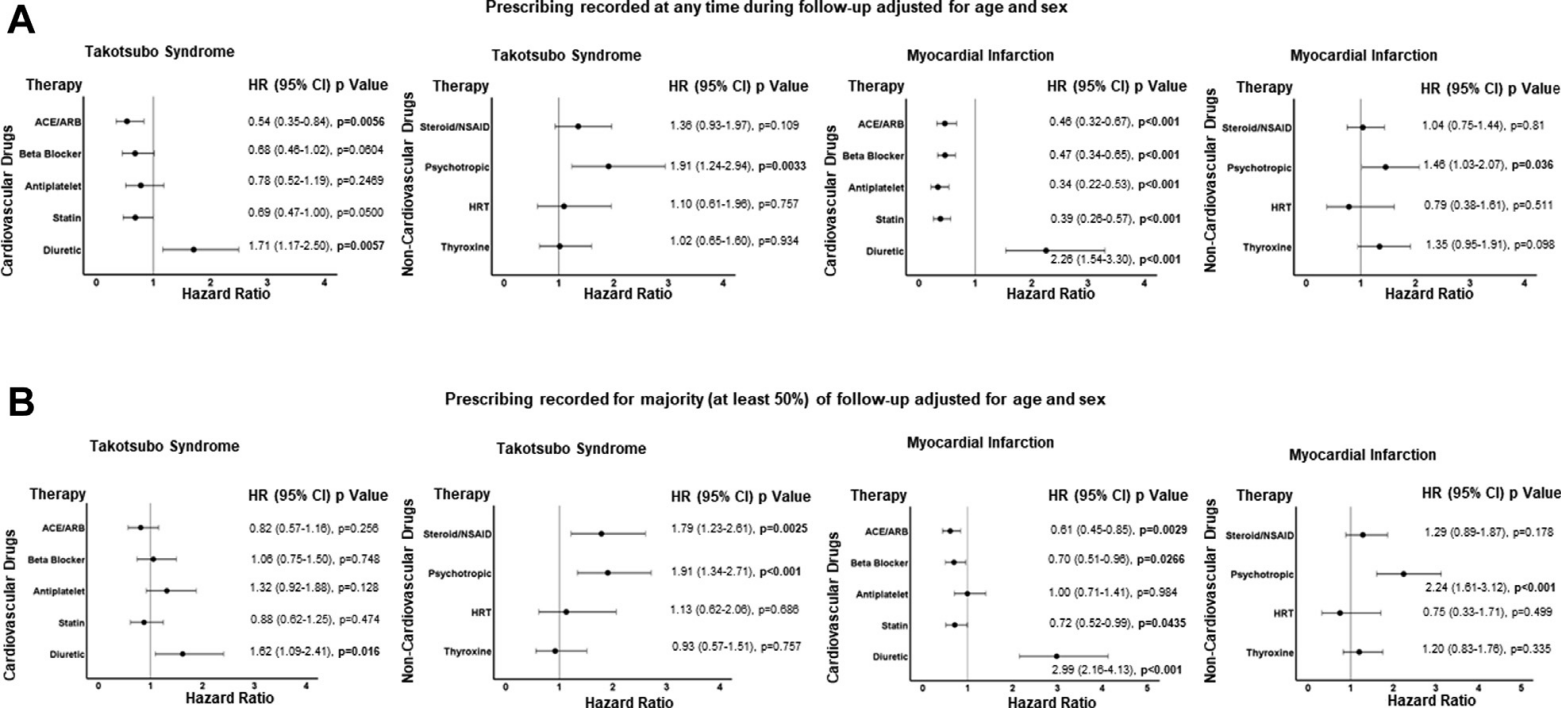
**Cardiovascular and Noncardiovascular Medications and Mortality** Patients with takotsubo syndrome (left panels) or myocardial infarction (right panels) mortality according to cardiovascular or noncardiovascular medication prescribed during follow-up in a binary analysis (A) or when medication was prescribed for the majority (at least 50%) of their follow-up duration (B). ACE = angiotensin-converting enzyme; ARB = angiotensin receptor blocker; HRT = hormone replacement therapy; NSAID = nonsteroidal anti-inflammatory drugs.

The only cardiovascular therapy associated with lower mortality in patients with takotsubo syndrome was angiotensin-converting enzyme inhibitor or angiotensin receptor blocker therapy (*P* = 0.0056). In contrast, angiotensin-converting enzyme inhibitor, angiotensin receptor blocker, beta-blocker, antiplatelet, and statin therapies were all associated with improved survival in patients with myocardial infarction (Figure 2A). Diuretic therapy was associated with worse outcomes in both patient groups (*P* = 0.0057 and *P* < 0.001, respectively). For noncardiovascular medications, psychotropic therapy was associated with increased rates of death in patients with takotsubo syndrome (HR: 1.91; 95% CI: 1.24-2.94; *P* = 0.003) and those with myocardial infarction (HR: 1.46; 95% CI: 1.03-2.07; *P* = 0.038). Adjusted analyses (age, sex, ST-segment elevation status, coronary artery disease, LV ejection fraction with or without diuretic medication) are displayed in Supplemental Figures 3A and 4A, respectively. Supplemental Figure 1 shows histograms of prescribing for each year of follow-up for all cardiovascular and noncardiovascular medications, whereas Supplemental Figure 2 shows the numbers of patients who died while receiving or not receiving each type of medication.

#### Prescribing recorded for the majority (at least 50%) of time during follow-up

No cardiovascular therapy remained associated with improved survival in patients with takotsubo syndrome. Angiotensin-converting enzyme inhibitor, angiotensin receptor blocker, beta-blocker, and statin therapies remained associated with improved survival in patients with myocardial infarction (Figure 2B). Diuretic therapy remained associated with worse outcomes in patients with either takotsubo syndrome or myocardial infarction (*P* = 0.016 and *P* < 0.001, respectively) as did psychotropic therapy (*P* < 0.001 and *P* < 0.001, respectively). Patients with takotsubo syndrome requiring chronic anti-inflammatory medication also had higher mortality (*P* = 0.002). Adjusted analyses (age, sex, ST-segment elevation status, coronary artery disease, LV ejection fraction with or without diuretic medication) are displayed in Supplemental Figures 3B and 4B.

## Discussion

Using comprehensive national data sets with robust matching of cases and controls, we have shown that patients with takotsubo syndrome have a substantially reduced survival compared to the general population that is comparable to patients with myocardial infarction and attributable to an excess of particularly cardiovascular but also noncardiovascular deaths. Survival is associated with prescribing profiles including the use of both cardiovascular and noncardiovascular therapies, but unlike in myocardial infarction, cardiovascular medications were not consistently associated with better long-term survival. These findings may help to lay the foundations for further exploration of potential mechanisms and treatments of this increasingly recognized and potentially fatal condition (Central Illustration).

**Central Illustration:**
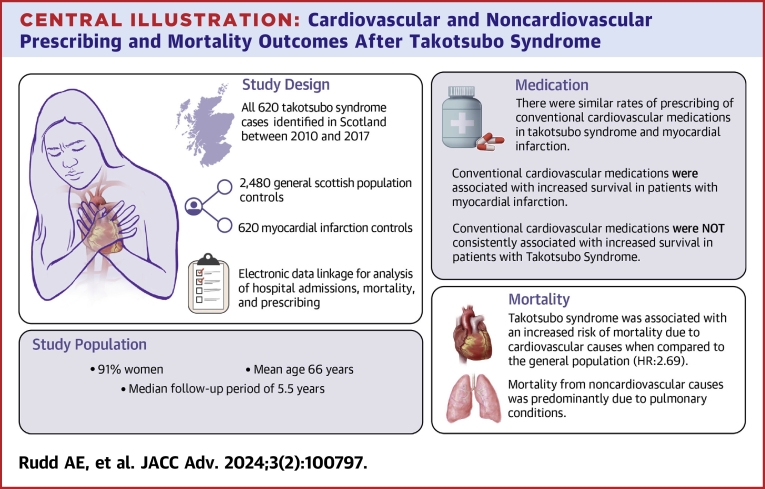
**Cardiovascular and Noncardiovascular Prescribing and Mortality Outcomes After Takotsubo Syndrome** The first report from the Scottish Takotsubo Registry, a retrospective case-control investigation reporting on the cardiovascular and noncardiovascular prescribing and mortality outcomes after acute takotsubo syndrome compared to acute myocardial infarction and general Scottish population controls.

Our data set has several unique and distinctive advantages over other contemporaneous registries.^6^^,^^7^^,^^14^^,^^15^ This is the first unselected comprehensive nationwide data set comprising all cases diagnosed with takotsubo syndrome and is therefore less susceptible to case ascertainment or selection biases. Diagnoses of both takotsubo syndrome and myocardial infarction^11^ were independently checked and adjudicated by their respective study investigators, ensuring precision of the diagnosis. The 2 control populations were matched using national data sets and key demographic variables. The general population is the ideal benchmark as it is representative of the expected survival for the geographic, health care, and national socioeconomic status. Moreover, the electronic data linkage of outcomes and prescriptions is a unique resource which overcomes many of the shortfalls associated with patient follow-up, as the only data loss pertains to cases who have emigrated or were not Scottish residents when they were admitted to a Scottish hospital with their index event.

To date, there has been major uncertainty regarding the causes of long-term death in takotsubo syndrome with some suggesting a major role of neuropsychiatric disorders or cancer.^5^^,^^16^^,^^17^ Our data show a major role of cardiovascular and pulmonary causes, with noncardiovascular rates of death being similar to those with myocardial infarction. The cardiovascular mortality in patients with takotsubo syndrome was mostly due to heart failure causes. Indeed, we have previously demonstrated that after the “recovery” from the acute episode, a proportion of patients evolve toward a heart failure with preserved ejection fraction phenotype in the longer term.^18^^,^^19^ The striking association with pulmonary causes of death may in part relate to the association between takotsubo syndrome and chronic obstructive pulmonary disease.^20^

This work provides the first data regarding long-term medication use. Surprisingly, patients with takotsubo syndrome receive comparable rates of prescribing as patients with myocardial infarction, despite the absence of any clinical trials or recommendations in international guidelines to guide such therapies. We found that only inhibitors of the renin-angiotensin system were associated with lower mortality in patients with takotsubo syndrome which is consistent with previous data from the Intertak registry.^5^ Such observational data are subject to confounding by indication for the prescription of such medications although the effect of renin-angiotensin system inhibition cannot be attributed to comorbidities, such as hypertension, which were of a similar frequency between patient cohorts. In addition, the association with renin-angiotensin system inhibition was inconsistent and was not demonstrable in those taking this medication for the majority (≥50%) of their follow-up duration. In contrast, cardiovascular therapies were associated with the expected survival benefit in patients with myocardial infarction irrespective of long-term compliance with maintenance therapy. Thus, the effects of modern cardiovascular therapies on survival in patients with takotsubo syndrome are unclear and require prospective evaluation in randomized controlled trials. Overall, it appears that the search for appropriate life-saving medication after takotsubo syndrome is only beginning and has yet to be realized.

The requirement for symptom alleviating therapies, such as diuretic or anti-inflammatory medication, is associated with worse survival after takotsubo syndrome and this association was also seen with diuretic therapy for patients with myocardial infarction. This likely reflects the decreased survival of those who develop symptomatic heart failure. The harmful association with increased use of psychotropic medication is also interesting and further highlights the high rates of mental health conditions seen particularly in patients with takotsubo syndrome^1^ as well as those with myocardial infarction. There are several potential explanations for these associations including common responses to those with more severe illness or perhaps drug-related adverse effects. Finally, even though the rates of prescribing were lower than for other medications, hormone replacement therapy did not have any effect on outcome, a surprising finding given the theory that perimenopausal female hormone changes may have a predisposing role for this condition in women.

### Study limitations

First, the clinical characteristics of patients with takotsubo syndrome and myocardial infarction were collected as predefined by each study design, and complete alignment of all variables was not feasible. Second, the study population was identified when the diagnosis of takotsubo syndrome was less familiar to clinicians and the condition was still underdiagnosed. This was particularly the case in the earlier stages of the study period. As this study was conducted as a national data set of routinely collected data, not all desirable information is available, in particular, no indication why drugs have been prescribed or discontinued. This may result in the inclusion of treatment for other reasons potentially leading to imprecision of results. Finally, even though the prescribing information is complete for the entire follow-up duration, limited inferences can be made from non-randomized data, causality cannot be assumed, and randomized studies are required to address this knowledge gap.

PERSPECTIVES **COMPETENCY IN MEDICAL KNOWLEDGE:** Takotsubo syndrome is associated with an excess of both cardiovascular and noncardiovascular mortality which is dominated by cardiovascular and pulmonary causes.

**TRANSLATIONAL OUTLOOK 1:** There are no clear relationships to concomitant cardiovascular therapies suggesting the routine use of such treatments is of uncertain benefit.

**TRANSLATIONAL OUTLOOK 2:** Randomized controlled trials of therapeutic interventions are urgently needed to address this major unmet clinical need.

## Conclusions

Cardiovascular and pulmonary causes account for a major proportion of deaths after takotsubo syndrome, with cardiovascular medications showing only weak and inconsistent associations with survival. Further studies to identify the etiology and specific pathophysiology of takotsubo syndrome are urgently needed as well as randomized controlled trials of therapeutic interventions.

## Funding support and author disclosures

Dr Dawson has received Chief Scientist Office Scotland award CGA-16-4 and the 10.13039/501100000274BHF Research Training Fellowship (FS/RTF/20/30009, for Ms Amelia Rudd). All other authors have reported that they have no relationships relevant to the contents of this paper to disclose.
