# Supplementary material for: Cardiovascular and Noncardiovascular Prescribing and Mortality After Takotsubo Comparison With Myocardial Infarction and General Population
Source: JACC Adv. Author manuscript; Available in PMC 2024 May 21. (PMC7615966; doi:10.1016/j.jacadv.2023.100797)
Supplement: Supplementary material [file EMS196132-supplement-Supplementary_material.pdf]

Cardiovascular and Non-Cardiovascular Prescribing and Mortality in Patients with Takotsubo Syndrome

Supplemental Material

CONSORT diagram: Identification of study populations and follow-up

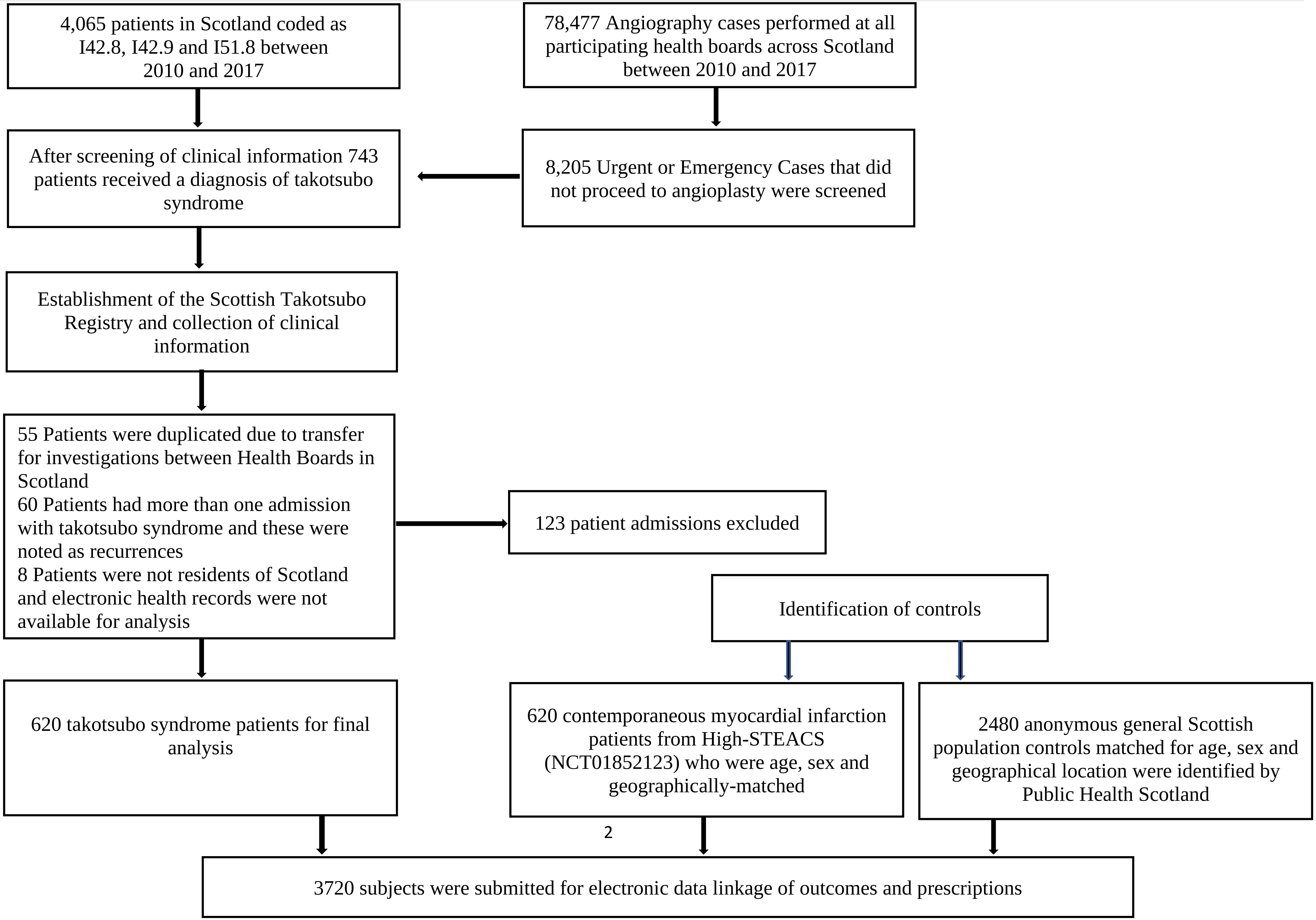

Supplemental Table 1. Specific diseases or causes of death in each system

	System	Specific diseases or causes of death in each system
1	Gastro-intestinal	Colitis, cirrhosis of the liver, alcohol liver disease, perforated bowel with/out peritonitis, biliary, fatty change of liver, volvulus, diseases of spleen
2	Infectious	Urinary, lung (pneumonia), otitis, upper or lower respiratory tract infection, osteomyelitis
3	Neurological	Motor Neuron Disease, Parkinson's, multiple sclerosis, seizures, organic amnesic syndrome, Huntington's
4	Vascular (peripheral)	Ischemic bowel, aortic aneurysm and aortic dissection of the descending aorta, Peripheral arterial disease, pulmonary embolism, venous ulcers, arteritis, vasculitis, pulmonary vessel diseases
5	Cardiac	Heart failure (acute or chronic), hypertensive heart disease, aortic stenosis/regurgitation, mitral stenosis/regurgitation, atrial fibrillation, any tachycardia or brady-arrhythmia, myocardial infarction, takotsubo recurrence, cardiomyopathy, infective endocarditis, pulmonary hypertension, pulmonary oedema, sudden cardiac death, death due to an acute cardiology procedure, death due to haemorrhage because of anticoagulation for a cardiac cause (valve replacement, atrial fibrillation, dual anti-platelet therapy), rheumatic heart disease, aortic dissection in context of aortic valve disease or proximal aortic/arch dissection, pericardium conditions
6	Natural/accident	Senility, old age, road traffic accident, accidental fall

7	Psychiatric	Anorexia, mental disorders due to alcohol, drug use, all psychiatric diagnoses
8	Pulmonary	Chronic obstructive pulmonary disease, pulmonary fibrosis, asthma, emphysema, interstitial lung disease
9	Renal	Nephritic, renal failure, chronic kidney disease
10	Endocrine	Diabetes, thyroid, pheochromocytoma
11	Cancer	Any cancer, anywhere
12	Cerebrovascular	Stroke, transient ischaemic attack, subarachnoid haemorrhage, intra-cerebral haemorrhage
13	Haemorrhage	Other cause, neither cardiovascular nor a stroke
14	Chronic inflammatory	Autoimmune conditions: systemic lupus erythematosus, scleroderma, immunoglobulin deficiencies, phlebitis, rheumatoid arthritis, Raynaud's, etc
15	Iatrogenic	Postprocedural disorders/complications
16	Dementia	
17	Other	Orthopaedic and musculo-skeletal, obstetric-gynaecology, dermatology, eye diseases, congenital conditions and any other non-life-threatening conditions (benign tumours, intolerances, blood disorders, hypercholesterolaemia)

**Supplemental Table 2. Cardiovascular causes of death**		
**Cardiovascular Cause of Death**	**Patients with Takotsubo Syndrome (n=47*)**	**Patients with Myocardial Infarction (n=85†)**
Heart Failure	25 (53.2%)	37 (43.5%)
Myocardial Infarction	13 (27.7%)	45 (52.9%)
Stroke	3 (6.4%)	1 (1.2%)
Sustained Ventricular Arrhythmia	4 (8.5%)	0
Cardiomyopathy	2 (4.3%)	
Endocarditis	0	2 (2.4%)

Data is presented as number (%)

*Number of patients with Takotsubo Syndrome who have died during follow-up period

†Number of patients with Myocardial Infarction who have died during follow-up period

**Supplemental Table 3. Baseline characteristics of takotsubo syndrome patients prescribed or not prescribed cardiovascular medications**				
	**Prescribed for majority of follow-up (>50%)**	**Prescribed at any time during follow-up**	**Not prescribed**	***p* =**
Age, years (mean ± SD)				
ACE/ARB	66 ± 11	63 ± 14	65 ± 14	0.030
Betablocker	66 ± 12	64 ± 13	66 ± 13	0.188
Antiplatelets	67 ± 11	65 ± 12	63 ± 14	0.004
Statin	66 ± 11	65 ± 11	64 ± 14	0.067
Diuretic	70 ± 10	68 ± 12	63 ± 12	<0.001
Sex (Female) (n (%))				
ACE/ARB	285 (92.2%)	164 (87.7%)	115 (92.7%)	0.173
Betablocker	261 (91.3%)	163 (89.6%)	140 (92.1%)	0.702
Antiplatelet Therapy	232 (88.9%)	163 (93.7%)	169 (91.4%)	0.227
Statin	266 (91.7%)	120 (93.0%)	178 (88.6%)	0.319
Diuretic	93 (92.1%)	151 (91.5%)	320 (90.4%)	0.838
Sex (Male) (n (%))				
ACE/ARB	24 (7.8%)	23 (12.3%)	9 (7.3%)	0.173
Betablocker	25 (8.7%)	19 (10.4%)	12 (7.9%)	0.702

Antiplatelets	29 (11.1%)	11 (6.3%)	16 (8.6%)	0.227
Statin	24 (8.3%)	9 (7.0%)	23 (11.4%)	0.319
Diuretic	8 (7.9%)	14 (8.5%)	34 (9.6%)	0.838
ST-Segment Elevation (n (%))				
ACE/ARB	138 (44.7%)	68 (36.4%)	53 (42.7%)	0.187
Betablocker	123 (43.0%)	63 (34.6%)	73 (48.0%)	0.040
Calcium Channel Blocker	30 (41.7%)	107 (42.1%)	122 (41.5%)	0.989
Antiplatelet Therapy	117 (44.8%)	67 (38.5%)	75 (40.5%)	0.391
Statin	121 (41.7%)	42 (32.6%)	96 (47.8%)	0.024
Diuretic	50 (49.5%)	73 (44.2%)	136 (38.4%)	0.104
Coronary Artery Disease (n (%))				
ACE/ARB	39 (14.1%)	19 (10.9%)	12 (11.7%)	0.583
Betablocker	39 (15.2%)	16 (9.6%)	15 (11.5%)	0.217
Antiplatelet Therapy	44 (18.6%)	16 (10.1%)	10 (6.3%)	0.001
Statin	46 (17.6%)	15 (12.3%)	9 (5.3%)	0.001
Diuretic	15 (16.9%)	18 (12.7%)	37 (11.5%)	0.398
Factor Increase 12 Hour Troponin (mean ± SD)				

ACE/ARB	165 ± 616	235 ± 1587	200 ± 771	0.845
Betablocker	256 ± 1386	107 ± 147	164 ± 696	0.469
Antiplatelet Therapy	260 ± 1453	91 ± 179	201 ± 676	0.389
Statin	150 ± 613	370 ± 1910	116 ± 171	0.167
Diuretic	130 ± 235	134 ± 215	234 ± 1319	0.642
LV Ejection Fraction <40% (n (%))				
ACE/ARB	179 (69.1%)	98 (64.1%)	50 (57.5%)	0.127
Betablocker	153 (65.4%)	101 (66.0%)	73 (65.2%)	0.988
Calcium Channel Blocker	35 (64.8%)	134 (64.4%)	158 (66.7%)	0.878
Antiplatelets	129 (61.1%)	89 (64.0%)	109 (73.2%)	0.056
Statin	153 (64%)	65 (60.2%)	109 (71.7%)	0.124
Diuretic	62 (74.7%)	94 (70.7%)	171 (60.4%)	0.019

All data are displayed as mean ± standard deviation and percentages

ACE=angiotensin-enzyme converting inhibitor

ARB=angiotensin receptor blocker

Supplemental Figure 1: Number of patients taking or not taking each type of medication during each year of study follow-up

Takotsubo Syndrome ACE/ARB

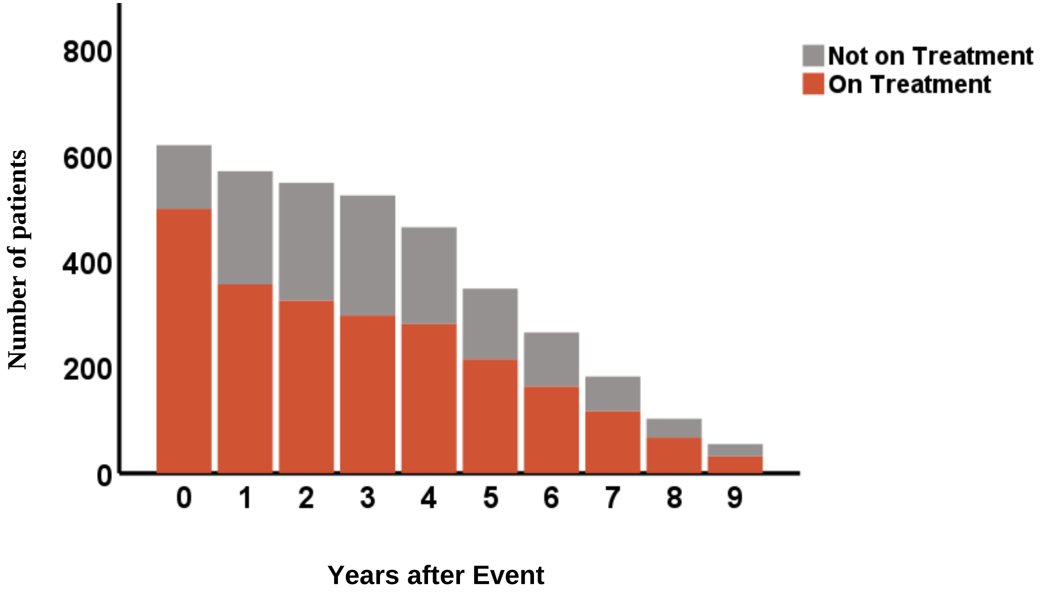

Myocardial Infarction ACE/ARB

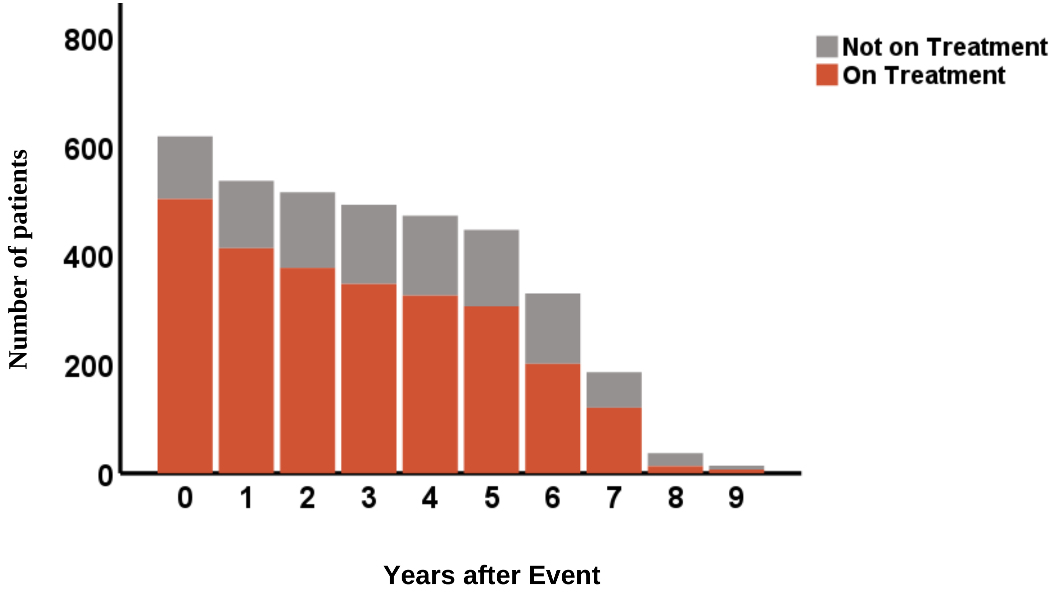

Takotsubo Syndrome Beta Blocker

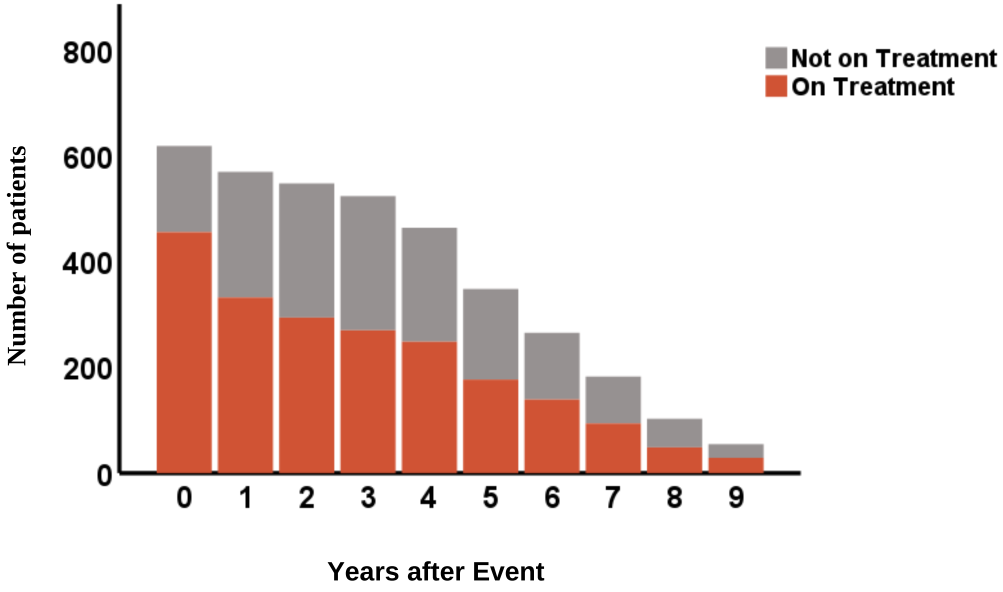

Myocardial Infarction Beta Blocker

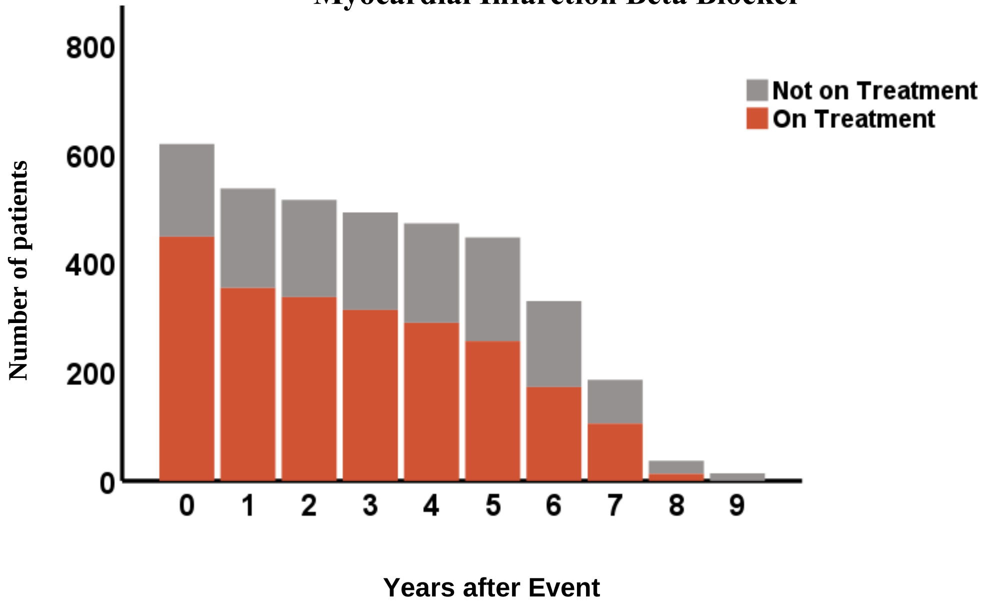

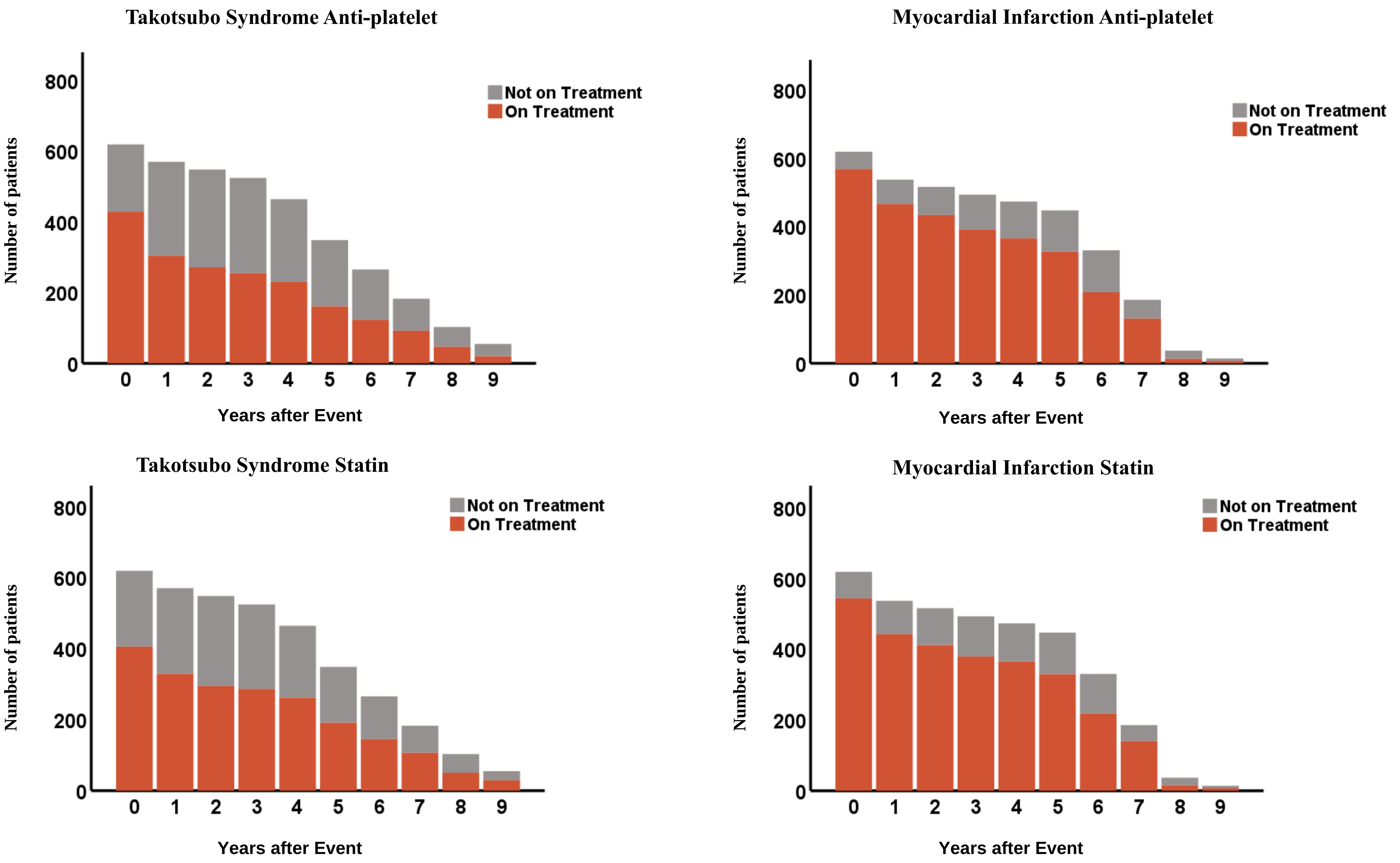
Takotsubo Syndrome Anti-platelet
Myocardial Infarction Anti-platelet
Takotsubo Syndrome Statin
Myocardial Infarction Statin
Number of patients
Years after Event
Not on Treatment
On Treatment
0
200
400
600
800
0 1 2 3 4 5 6 7 8 9

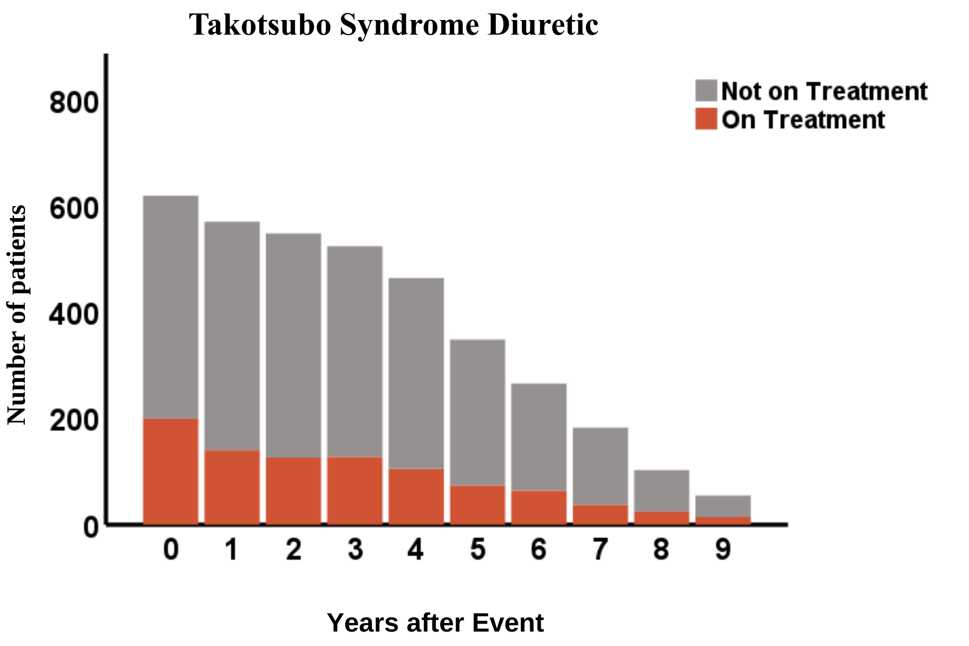
Takotsubo Syndrome Diuretic
Not on Treatment
On Treatment
Number of patients
800
600
400
200
0
0 1 2 3 4 5 6 7 8 9
Years after Event

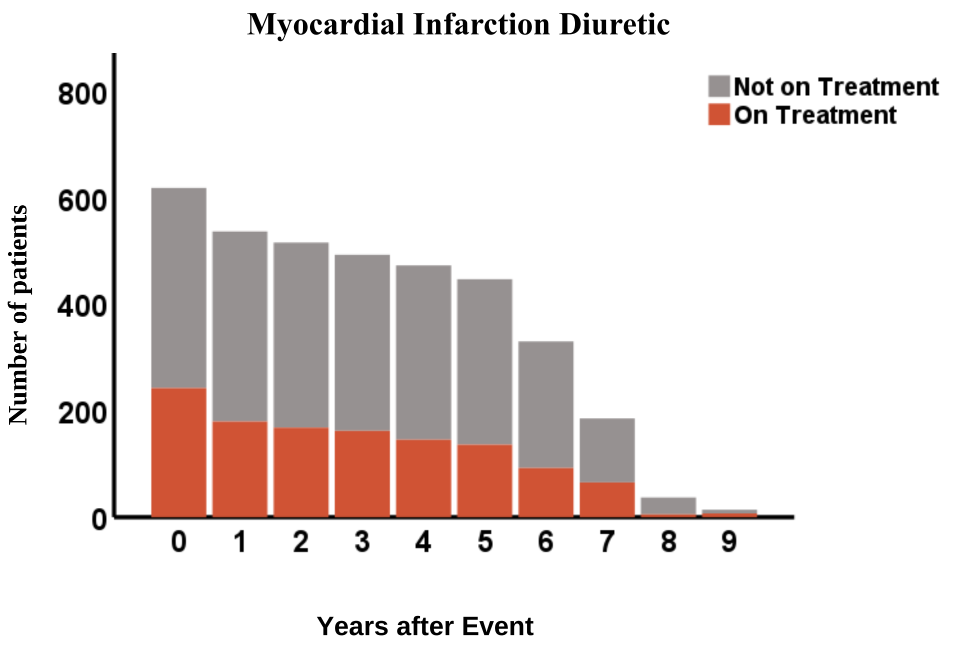
Myocardial Infarction Diuretic
Not on Treatment
On Treatment
Number of patients
800
600
400
200
0
0 1 2 3 4 5 6 7 8 9
Years after Event

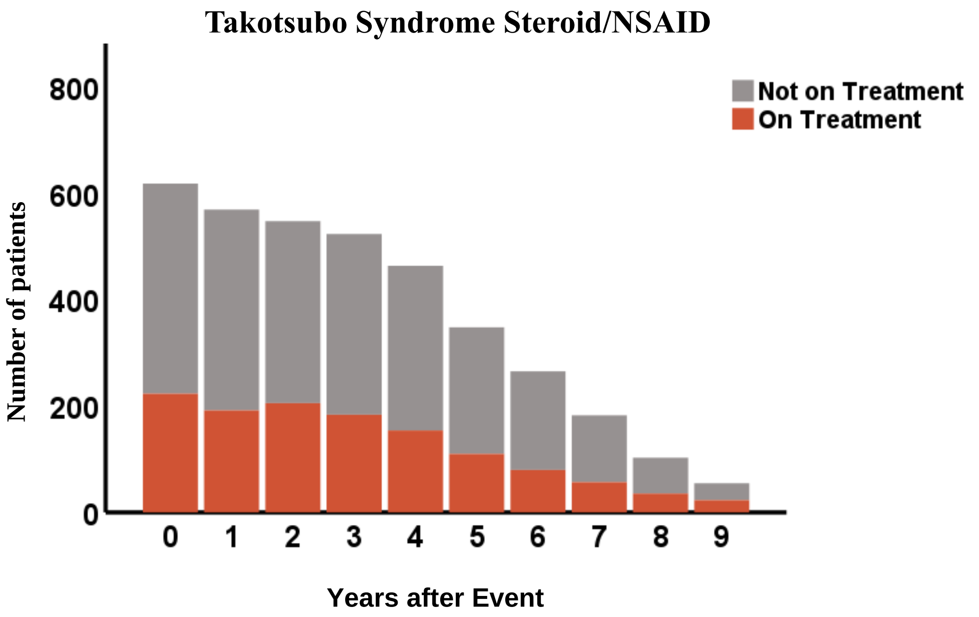
Takotsubo Syndrome Steroid/NSAID
Not on Treatment
On Treatment
Number of patients
800
600
400
200
0
0 1 2 3 4 5 6 7 8 9
Years after Event

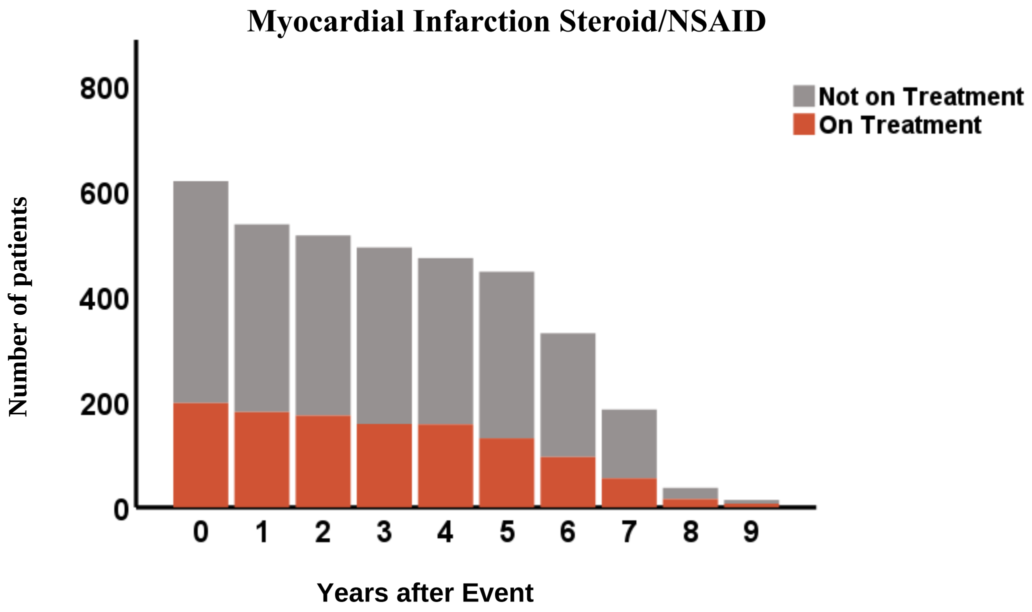
Myocardial Infarction Steroid/NSAID
Not on Treatment
On Treatment
Number of patients
800
600
400
200
0
0 1 2 3 4 5 6 7 8 9
Years after Event

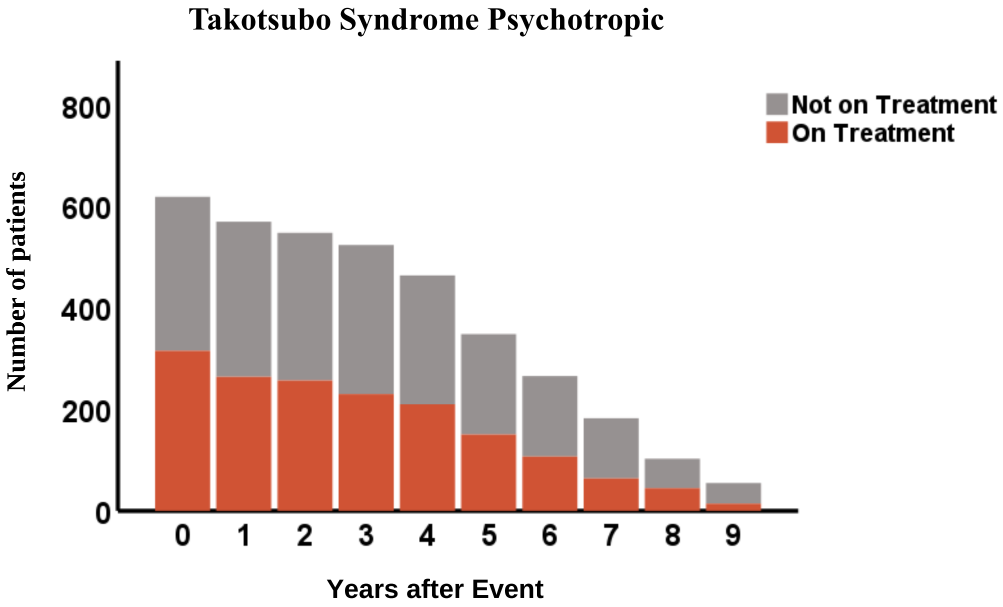
Takotsubo Syndrome Psychotropic
Not on Treatment
On Treatment
Number of patients
800
600
400
200
0
0
1
2
3
4
5
6
7
8
9
Years after Event

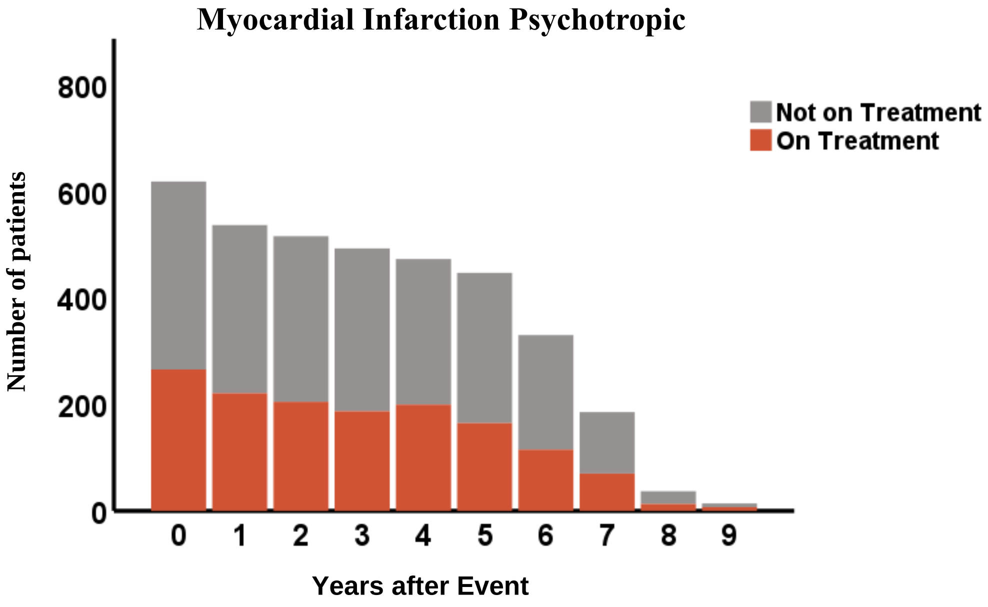
Myocardial Infarction Psychotropic
Not on Treatment
On Treatment
Number of patients
800
600
400
200
0
0
1
2
3
4
5
6
7
8
9
Years after Event

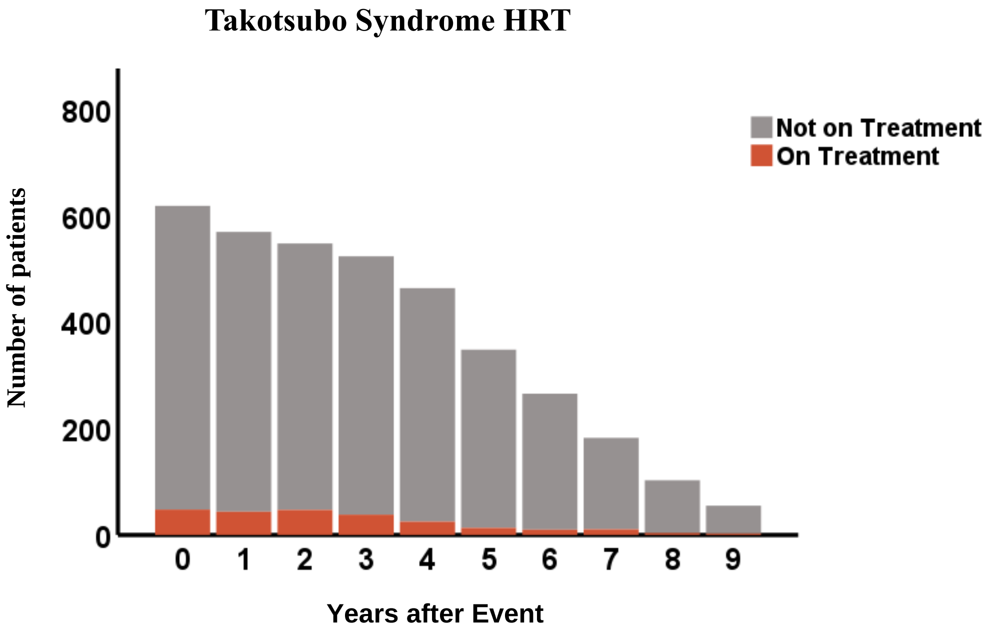
Takotsubo Syndrome HRT
Not on Treatment
On Treatment
Number of patients
800
600
400
200
0
0
1
2
3
4
5
6
7
8
9
Years after Event

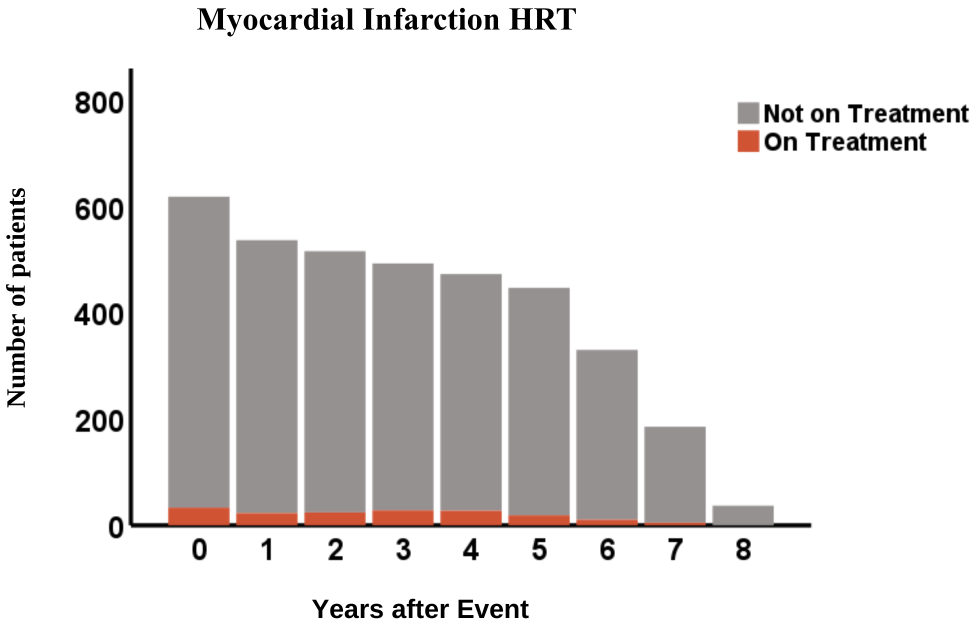
Myocardial Infarction HRT
Not on Treatment
On Treatment
Number of patients
800
600
400
200
0
0
1
2
3
4
5
6
7
8
Years after Event

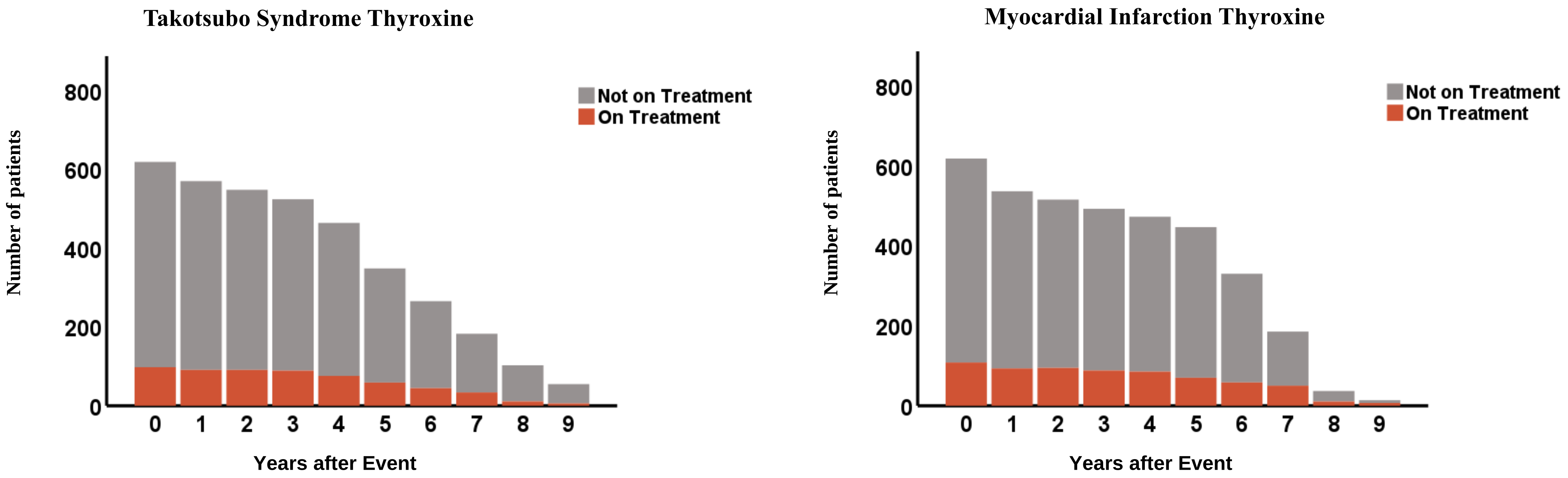

Takotsubo Syndrome Thyroxine
Number of patients
800
600
400
200
0
Not on Treatment
On Treatment
0 1 2 3 4 5 6 7 8 9
Years after Event
Myocardial Infarction Thyroxine
Number of patients
800
600
400
200
0
Not on Treatment
On Treatment
0 1 2 3 4 5 6 7 8 9
Years after Event

Supplemental Figure 2: Number of patients who died each year whilst receiving or not receiving each type of therapy

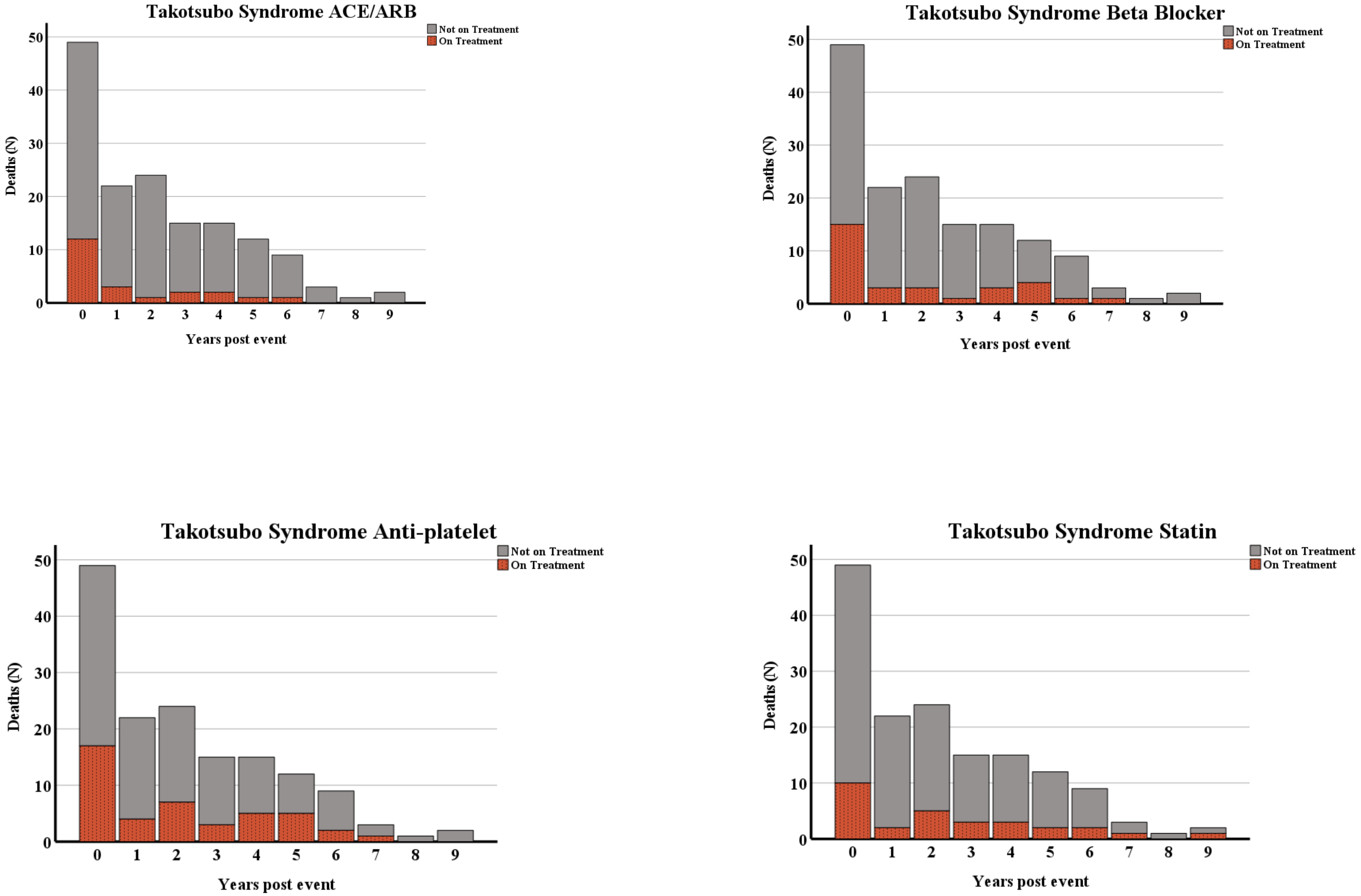

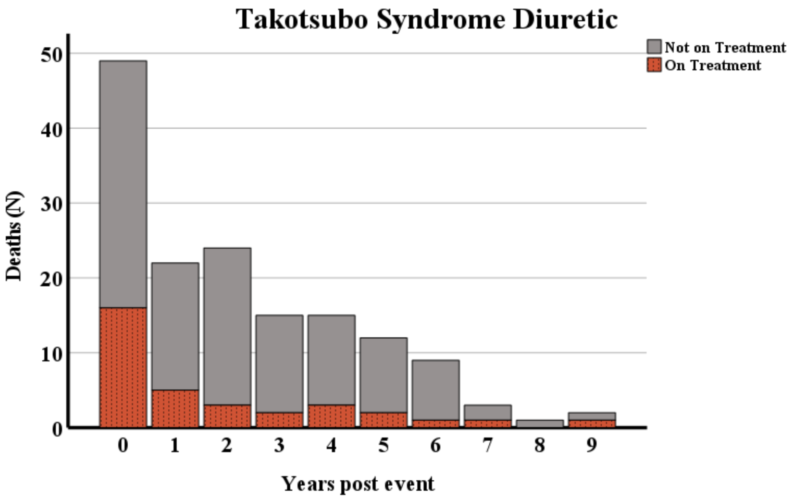
Takotsubo Syndrome Diuretic
Not on Treatment
On Treatment
Deaths (N)
50
40
30
20
10
0
0
1
2
3
4
5
6
7
8
9
Years post event

Supplemental Figure 3A

Prescribing recorded at any time during follow-up adjusted for age, sex, ST elevation status and coronary artery disease

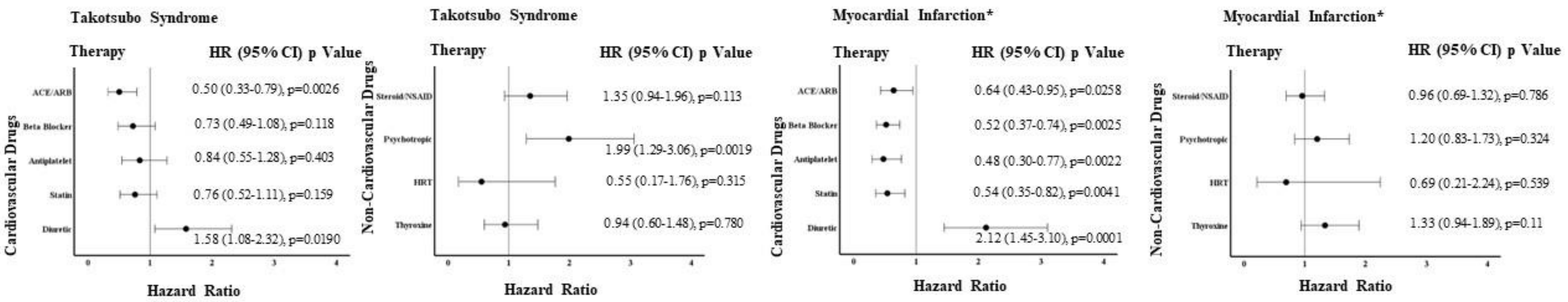

Supplemental Figure 3B

Prescribing recorded for majority (at least 50%) of follow-up adjusted for age, sex, ST elevation status and coronary artery disease

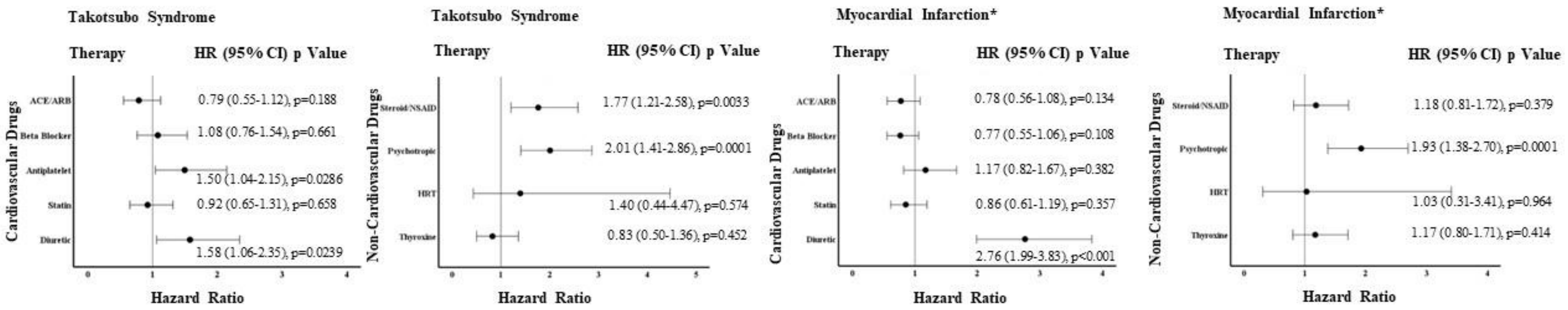

*Myocardial infarction was not adjusted for LV ejection fraction due to data not being collected for this cohort

Supplemental Figure 4A

Prescribing recorded at any time during follow-up adjusted for age, sex, ST elevation status, LV ejection fraction, coronary artery disease and diuretic medication

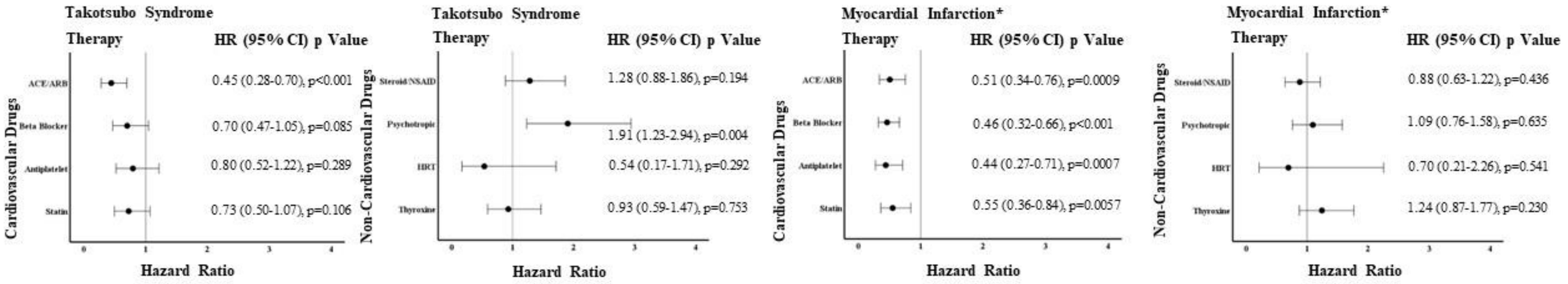

Supplemental Figure 4B

Prescribing recorded for majority (at least 50%) of follow-up adjusted for age, sex, ST elevation status, LV ejection fraction, coronary artery disease and diuretic medication

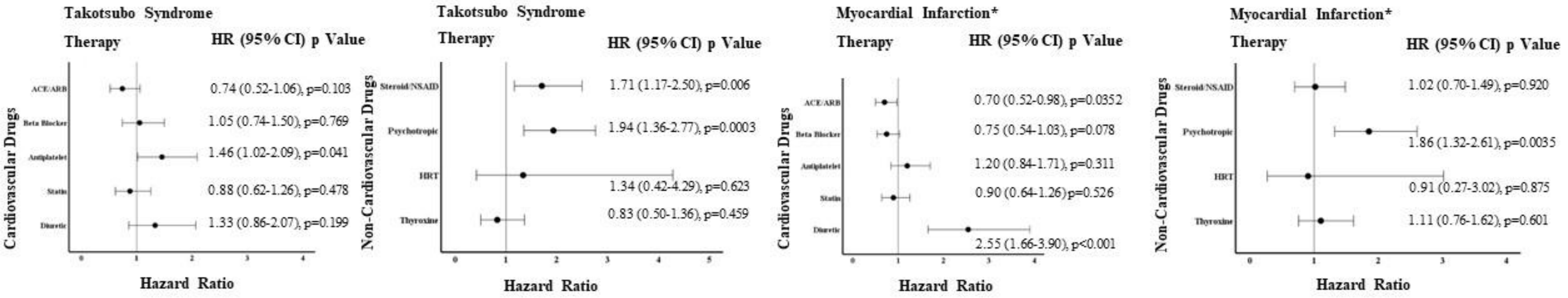

* Myocardial infarction was not adjusted for LV ejection fraction due to data not being collected for this cohort
